# PRESCO: an online tool for predicting severe pulmonary complications and survival after cancer surgery

**DOI:** 10.3389/fonc.2025.1705181

**Published:** 2026-01-07

**Authors:** Ke Luo, Yunyun Su, Lu Wang

**Affiliations:** 1 Department of Anesthesiology, Hunan Cancer Hospital, Changsha, China; 2 Department of Intensive Care Unit, Hunan Cancer Hospital, Changsha, China

**Keywords:** postoperative respiratory failure, pulmonary embolism, machine learning, cancer surgery, risk prediction

## Abstract

**Background:**

Severe pulmonary complications (SPCs) after cancer surgery have a significant impact on morbidity, mortality, and healthcare burden. Despite this, clinicians currently lack accurate and practical tools to predict the occurrence and survival outcomes of SPCs.

**Methods:**

We conducted a retrospective cohort study of cancer patients undergoing surgery at Hunan Cancer Hospital between June 2023 and June 2025. Two datasets were established (1): 434 patients (227 with SPCs and 207 controls) for predicting SPCs occurrence, and (2) 227 SPC patients with complete follow-up data for 28-day and 90-day survival prediction. Six supervised machine learning classifiers, including linear discriminant analysis (LDA), support vector machine (SVM), random forest (RDF), decision tree (DST), adaptive boosting (ADA), and extremely randomized trees (EXT), were developed. Hyperparameters were optimized using grid search with five-fold stratified cross-validation. Performance was assessed using testing sets by evaluating the Brier score, precision, recall, F1-score, and AUC. SHapley Additive exPlanations (SHAP) were used for model interpretability, and the finalized models were deployed as web-based applications.

**Results:**

For SPC occurrence prediction, the EXT model demonstrated the best performance (AUC = 0.813). For 28-day mortality prediction, EXT achieved the highest discrimination (AUC = 0.921), whereas RDF performed best for 90-day mortality (AUC = 0.899). SHAP analysis identified preoperative hypertension, ECOG score, and intraoperative blood loss as the most influential predictors of SPC occurrence. Postoperative SOFA scores, APACHE II scores, and blood urea nitrogen (BUN) were key predictors of 28-day and 90-day mortality. PRESCO (https://presco.streamlit.app/), an online tool, provides real-time prediction of SPC and survival outcomes.

**Conclusions:**

We developed and validated machine learning models that accurately predict the occurrence and survival of SPCs in cancer patients after surgery. By deploying the online tool, clinicians can easily access it and utilize its functions to perform personalized risk stratification and guide perioperative decisions in oncology.

## 1 Introduction

Postoperative complications remain a major cause of morbidity and mortality in cancer patients undergoing surgical treatment (1). Among these, postoperative respiratory failure (PRF) and pulmonary embolism (PE) are two of the most severe pulmonary complications (SPCs). Approximately 5% of patients with solid tumors develop PRF, while PE occurs in up to 10% of cancer patients following surgery (2). PRF is defined as the inability to maintain adequate gas exchange after surgery, and patients often need mechanical ventilation. PE is defined as thromboembolic obstruction of the pulmonary circulation. Both complications could prolong hospitalization, increase healthcare costs, and impair short-term survival (3). For instance, the in-hospital mortality of PRF is reported to range between 25% and 40% (4). Therefore, early identification of patients at high risk for SPCs and accurate prediction of outcomes in SPC patients are essential for perioperative management and individualized care.

Current predictive approaches fall into two categories: traditional scoring (e.g., SPORC-2, ARISCAT and nomogram) (5) and machine learning–based models. Scoring systems and nomograms offer excellent convenience but are often limited in accuracy. For example, one study developed a nomogram to predict postoperative venous thromboembolism in patients with stage IA lung cancer and reported an AUC of 0.791 in validation cohorts (6). In contrast, machine learning approaches have demonstrated superior performance. A study using eight easily extractable electronic health record (EHR) variables achieved an AUC of 0.93 for predicting PRF (7). The RESPIRE model similarly showed an AUC of 0.93 (8). Despite their high accuracy, machine learning tools are often difficult to implement in real-world clinical practice. Thus, there is an urgent need for predictive tools that combine the accuracy of machine learning with the usability required for routine clinical application.

In this study, we developed and validated PRESCO (Prediction of Respiratory failure, Embolism, and Survival after Cancer Operation), a machine learning–based online tool designed to address two critical clinical tasks. The first was to predict the occurrence of SPCs in cancer patients undergoing surgery. The second was to predict 28-day and 90-day mortality among those who developed SPCs. We systematically evaluated multiple models using perioperative and postoperative features. The best-performing models were deployed as user-friendly, browser-based applications for real-time clinical decision support. Our goal was to provide clinicians with accurate and interpretable risk stratification tools that enable early identification of high-risk patients and improve postoperative management in surgical oncology.

## 2 Methods

### 2.1 Data source and study design

This retrospective cohort study was conducted using the EHR data of cancer patients who underwent surgery at Hunan Cancer Hospital between June 1, 2023, and June 1, 2025. To protect patient privacy, all identifiable information was anonymized. The primary outcome was the occurrence of severe postoperative pulmonary complications (SPCs), defined as postoperative respiratory failure (PRF) or pulmonary embolism (PE). PRF is diagnosed when PaO_2_< 60 mmHg with or without PaCO_2_> 50 mmHg, or when FiO_2_ ≠ 21% and PaO_2_/FiO_2_ ≤ 300 mmHg. PE is diagnosed by typical clinical manifestations such as acute dyspnea, chest pain, or unexplained hypoxemia, combined with imaging confirmation from computed tomography pulmonary angiography. All suspected cases of PRF and PE were independently reviewed by two senior attending intensivists, and any discrepancies were resolved through consensus discussion. The final diagnoses were subsequently confirmed by a multidisciplinary team (MDT) to ensure diagnostic accuracy and consistency. The postoperative observation window for event capture was restricted to within 7 days after surgery, consistent with international definitions of postoperative pulmonary complications, to ensure temporal relevance to perioperative factors.

Two datasets were used for model development: one for predicting the occurrence of SPC in cancer patients after surgery, and the other for predicting survival outcomes among patients diagnosed with SPC. For the first dataset, patients were included if they met the following criteria (1): histologically confirmed diagnosis of cancer; (2) underwent surgical treatment; and (3) had sufficient perioperative data available. Exclusion criteria were: (1) age < 18 years and (2) preoperative severe pulmonary dysfunction requiring mechanical ventilation. A total of 227 patients who developed SPC were identified, and 207 patients without SPC were randomly selected as controls. Thus, a dataset of 434 patients was obtained. For the second dataset, only patients who developed SPC were included. The same inclusion and exclusion criteria were applied, with the additional requirement of available follow-up data on survival at 28 and 90 days postoperatively. Finally, 227 cancer patients with SPC and complete follow-up data were included in the survival analysis.

Missing values were handled before model development. For continuous variables, missing data were imputed using the median value calculated from the training set. For categorical variables, the most frequent category in the training set was used for imputing missing values. Imputation parameters derived from the training data were then applied to the testing data to avoid information leakage. A summary of the proportion of missing values for each variable in the dataset used to predict SPC occurrence is provided in **Supplementary Table 1**. A summary of the proportion of missing values for each variable in the dataset used to predict 28-day and 90-day mortality outcomes is provided in **Supplementary Table 2**.

### 2.2 Features for predicting occurrence of SPCs in cancer patients

The prediction task in this section was to determine whether a cancer patient would develop SPCs after surgery. A total of 19 preoperative predictors from multiple domains were included: (1) demographics: age, sex, height, and weight; (2) tumor-related characteristics: tumor type (e.g., lung, bladder, colon), T stage, N stage, and M stage; (3) comorbidities: hypertension, diabetes mellitus, coronary artery disease, and stroke; (4) preoperative evaluation and nutritional status: American Society of Anesthesiologists (ASA) score, Eastern Cooperative Oncology Group (ECOG) performance status, Geriatric Nutritional Risk Index (GNRI), preoperative serum albumin, and preoperative FEV1/FVC ratio; (5) perioperative factors: duration time of surgery and intraoperative blood loss. These variables were used to build models predicting the occurrence of SPCs.

### 2.3 Features for predicting 28-day and 90-day mortality in patients with SPCs

For patients diagnosed with SPC, a total of 56 predictors were incorporated into the prognostic models for predicting 28-day and 90-day mortality. These comprised: (1) disease type (PRF or PE); (2) demographics: age, sex, height, and weight; (3) tumor characteristics: tumor type, T stage, N stage, and M stage; (4) comorbidities: hypertension, diabetes mellitus, coronary artery disease, and stroke; (5) preoperative evaluation and nutritional/functional status: ASA score, ECOG performance status, GNRI, preoperative serum albumin, and FEV1/FVC ratio; (6) perioperative factors: duration of surgery and intraoperative blood loss; (7) postoperative respiratory support and gas exchange: mechanical ventilation time, postoperative PaO_2_, postoperative PaCO_2_, postoperative pH, Fraction of Inspired Oxygen (FiO_2_), PaO_2_/FiO_2_ ratio, ventilation mode, and Positive End-Expiratory Pressure (PEEP) level; (8) postoperative clinical status within 48 hours: SOFA score, APACHE II score, mean arterial pressure, systolic and diastolic blood pressure, heart rate, and blood lactate level; (9) postoperative laboratory findings within 48 hours: WBC, PCT, CRP, hemoglobin, platelet count, creatinine, blood urea nitrogen, postoperative serum albumin, PT, INR, APTT, blood glucose, bicarbonate, sodium, and potassium; and (10) postoperative complications and events: infection, delirium, acute kidney injury, arrhythmia, deep vein thrombosis, and use of sedative medications. These predictors comprehensively captured demographic, oncological, perioperative, and postoperative characteristics and were used to develop survival prediction models.

### 2.4 Feature selection, hyperparameter optimization, and model evaluation

From a clinical perspective, constraining the model to a limited number of routinely available and physiologically meaningful variables improves interpretability and facilitates implementation in real-world settings. Indeed, several validated online medical prediction tools incorporate approximately 10–15 features to maintain a balance between predictive performance and practical usability (9–11). Variable importance was evaluated using the random forest algorithm in the training set, and the top 12 influential predictors were retained for model development. This feature selection strategy effectively reduced model complexity while aligning with our goal of creating a clinically accessible and user-friendly predictive tool. Subsequently, six supervised machine learning algorithms were implemented with scikit-learn, including linear discriminant analysis (LDA), support vector machine (SVM), random forest (RDF), decision tree (DST), adaptive boosting (ADA), and extremely randomized trees (EXT), to develop predictive models.

Stratified random sampling was applied to divide the cohort into a training set (70%) and a testing set (30%), maintaining the original distribution of the outcome variable. The primary optimization criterion was the F1 score. The best-performing hyperparameters were retained for final model evaluation. For LDA, both svd and lsqr solvers were tested, with shrinkage options considered for the lsqr setting. The SVM was tuned by varying the regularization parameter C between 0.5 and 2 and the kernel coefficient gamma using either scale or auto. The RDF model was optimized over the number of trees, with 200 and 500 estimators considered, as well as maximum tree depths of none, 5, or 10, and minimum samples per leaf of 1 or 3. For the DST, maximum depths of none, 5, or 10 were explored, together with minimum split sizes of 2 or 5 and minimum samples per leaf of 1 or 3. ADA was tuned with 200 or 500 estimators and learning rates of 0.05, 0.1, or 0.5. EXT was optimized with 200 or 500 estimators, maximum depths of none, 5, or 10, and minimum samples per leaf of 1 or 3.

Final models, retrained on the entire training dataset with optimized hyperparameters, were evaluated on the testing cohort. To quantify the robustness and uncertainty of model performance estimates, a non-parametric bootstrap procedure with 100 resamples of the testing set was conducted (sampling with replacement, each resample matching the original testing set size). For each bootstrap iteration, the area under the receiver operating characteristic curve (AUC), recall, precision, F1-score, and Brier score were recalculated. Pairwise comparisons between models were conducted using the Wilcoxon rank-sum test, a non-parametric method for comparing the distribution of repeated model performance metrics. A two-sided p-value<0.05 was considered statistically significant. The mean values and corresponding 95% confidence intervals derived from the bootstrap distributions were used to assess the stability of model performance.

To compare the predictive capabilities of different feature configurations, mean AUC values were used to contrast the full-feature and reduced-feature models. For the best-performing model (with the highest AUC), sensitivity, specificity, positive predictive value (PPV), and negative predictive value (NPV) were computed across multiple probability thresholds. Model calibration was further evaluated using calibration plots, and clinical utility was assessed through decision-curve analysis (DCA). Finally, subgroup analyses were performed for patients with lung cancer versus those with non-lung cancer, and for those with ASA class II versus class III, to evaluate AUC values within these clinically relevant strata.

### 2.5 Model interpretation with SHAP

Model interpretability was further examined using SHapley Additive exPlanations (SHAP) to complement the impurity-based feature importance. SHAP is a game-theoretic approach that decomposes the prediction of a machine learning model into additive contributions of each feature. For each observation in the independent test set, SHAP values were computed using the TreeSHAP algorithm, which is specifically optimized for tree-based ensembles. These values quantify the marginal contribution of each feature to the model’s prediction relative to a baseline expectation. Summary plots were generated to visualize the global distribution of SHAP values across all features and the direction of their effects on the prediction outcome.

### 2.6 Web deployment of the predictive tool

A browser-based application, PRESCO (Prediction of Respiratory failure, Embolism, and Survival after Cancer Operation), was developed using Streamlit to provide real-time predictions of severe pulmonary complications and short-term mortality following cancer surgery. The platform consists of three modules: prediction of SPC occurrence, 28-day mortality, and 90-day mortality. Each interface accepts 12 selected predictive variables and executes the finalized machine learning model with optimized hyperparameters. The results are displayed as predicted probabilities accompanied by corresponding class decisions.

PRESCO is intended for healthcare professionals involved in perioperative care and is provided for research and educational purposes only. It should not replace professional medical judgment or serve as a diagnostic device. To protect user privacy, no identifiable patient data is collected, stored, or transmitted; all computations occur locally within the user’s browser under HTTPS encryption. Preliminary usability testing with anesthesiologists and intensivists indicated that the interface was intuitive, required less than one minute to complete, and presented results clearly and interpretably.

## 3 Results

### 3.1 Patient characteristics

**Figure 1:**
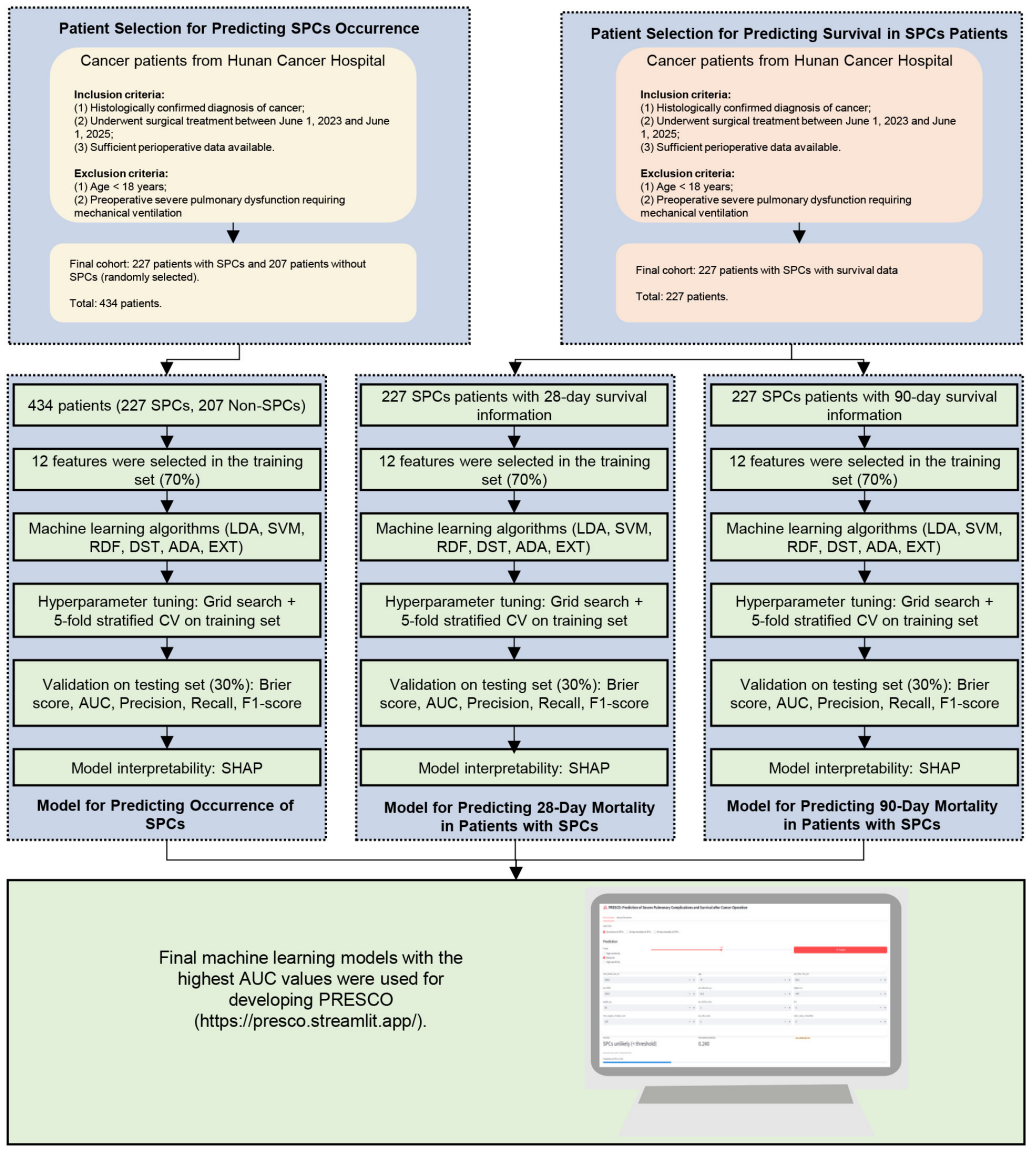
The workflow of this research.

The workflow of this research is shown in **Figure 1**. The occurrence cohort consisted of 434 patients, including 227 who developed SPCs after surgery and 207 who remained complication-free, serving as controls. This cohort was randomly divided into a training set (70%) and a testing set (30%). Baseline demographic and clinical characteristics of the two sets are summarized in **Supplementary Table 3**. The 19 collected variables included demographics, tumor-related characteristics, comorbidities, preoperative evaluation and nutritional status factors, and perioperative factors. No significant differences were observed between the training and testing sets across these variables except hemoglobin (all p-values > 0.05). In the training set, the mean age was 60.4 years. Lung cancer was the most common cancer type. The major comorbidities were hypertension (18.1%), diabetes mellitus (6.91%), and coronary artery disease (3.62%).

For the survival analysis of 28-day mortality, 227 SPC patients with complete follow-up data were included. The cohort (**Supplementary Table 4**) was randomly divided into a training set (70%) and a testing set (30%). In the training set, the mean age was 61.6 years. 44.7% of patients had PRF, and 55.3% had PE. At 28 days, 56.6% of patients were alive, whereas 43.4% had died. For the survival analysis of 90-day mortality, 227 SPC patients with complete follow-up data were included. The cohort (**Supplementary Table 5**) was randomly divided into a training set (70%) and a testing set (30%). In the training set, the mean age was 61.9 years. 46.5% of patients had PRF, and 53.5% had PE. At 90 days, 47.2% of patients were alive, whereas 52.8% had died.

### 3.2 Model performance for predicting SPCs occurrence

**Figure 2:**
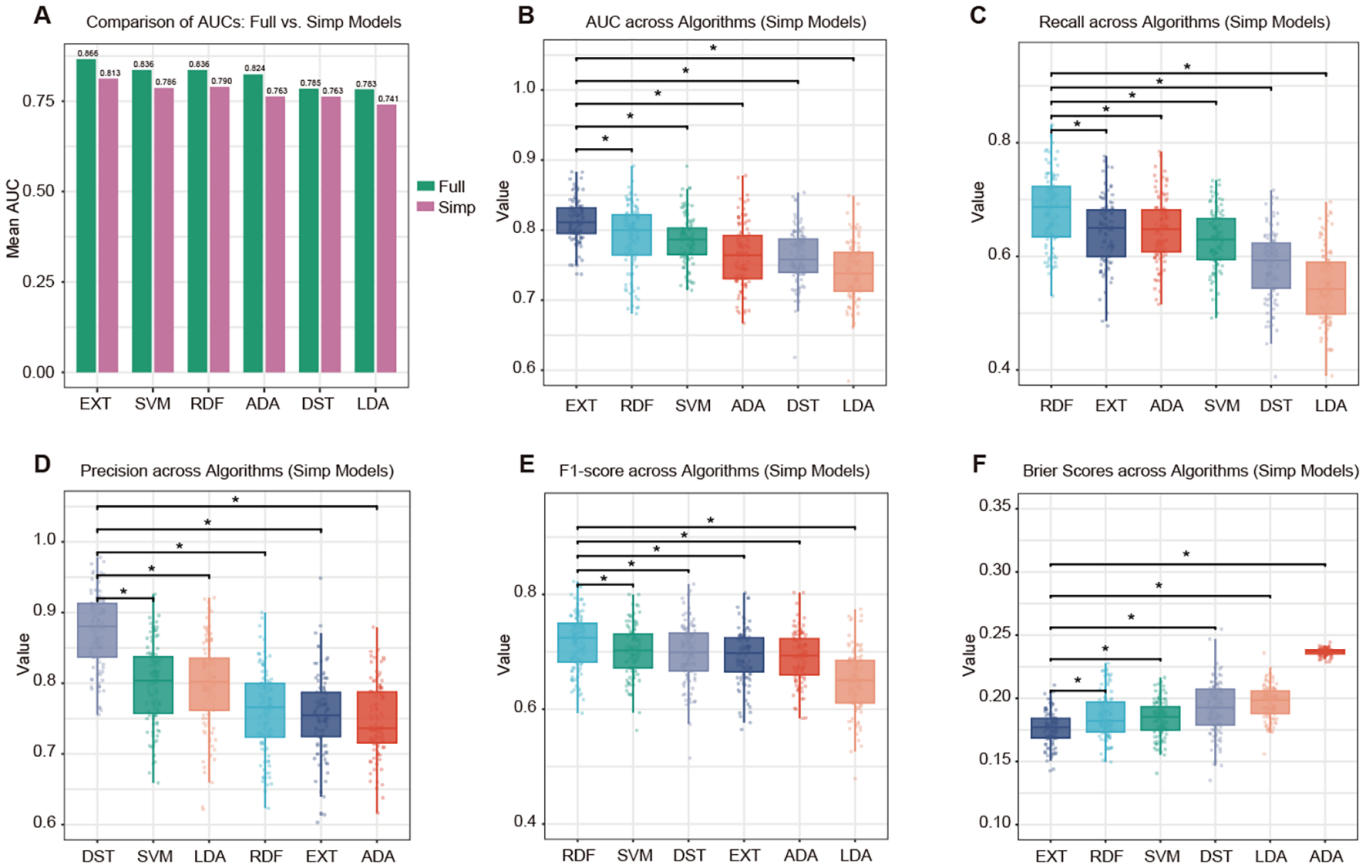
Model performance for predicting SPC occurrence. **(A)** Comparison of mean AUC values between models trained with all 19 available variables (Full) and those trained with the top 12 selected variables (Simp). Performance metrics of the simplified models across six machine learning algorithms, including linear discriminant analysis (LDA), support vector machine (SVM), random forest (RDF), decision tree (DST), adaptive boosting (ADA), and extremely randomized trees (EXT). Boxplots display the distributions of **(B)** AUC, **(C)** recall, **(D)** precision, **(E)** F1-score, and **(F)** Brier score derived from 100 bootstrap iterations. Horizontal lines denote statistically significant pairwise differences (*represents p-value < 0.05).

Feature importance was evaluated in the training dataset using the random forest algorithm, and the top 12 predictors were selected for model development. Six machine learning classifiers were trained and evaluated using either all 19 available variables (model-full) or the top 12 selected variables (model-simp). On the testing set, the model-full generally demonstrated slightly higher predictive performance than the simplified version. For example, the EXT model achieved an AUC of 0.866 in model-full compared with 0.813 in model-simp (**Figure 2A**). Nevertheless, restricting the model to 12 routinely available and physiologically meaningful predictors enhanced interpretability and facilitated clinical implementation. Among all algorithms, tree-based methods, including EXT and RDF, outperformed the other classifiers in overall discrimination and calibration. Specifically, EXT yielded the highest mean AUC of 0.813 (95% CI: 0.807–0.819; **Figure 2B**), RDF achieved the best recall (0.681, 95% CI: 0.669–0.694; **Figure 2C**), DST had the highest precision (0.873, 95% CI: 0.863–0.884; **Figure 2D**), RDF showed the best F1-score (0.717, 95% CI: 0.707–0.727; **Figure 2E**), and EXT obtained the lowest Brier score (0.176, 95% CI: 0.174–0.179; **Figure 2F**). With increasing probability thresholds, the model shifted from a highly sensitive but less specific mode to a more conservative and precise prediction pattern (**Supplementary Table 6**). At moderate thresholds (0.40–0.60), the model achieved a balanced diagnostic performance, maintaining both reasonable sensitivity (0.52–0.85) and specificity (0.61–0.87).

**Figure 3:**
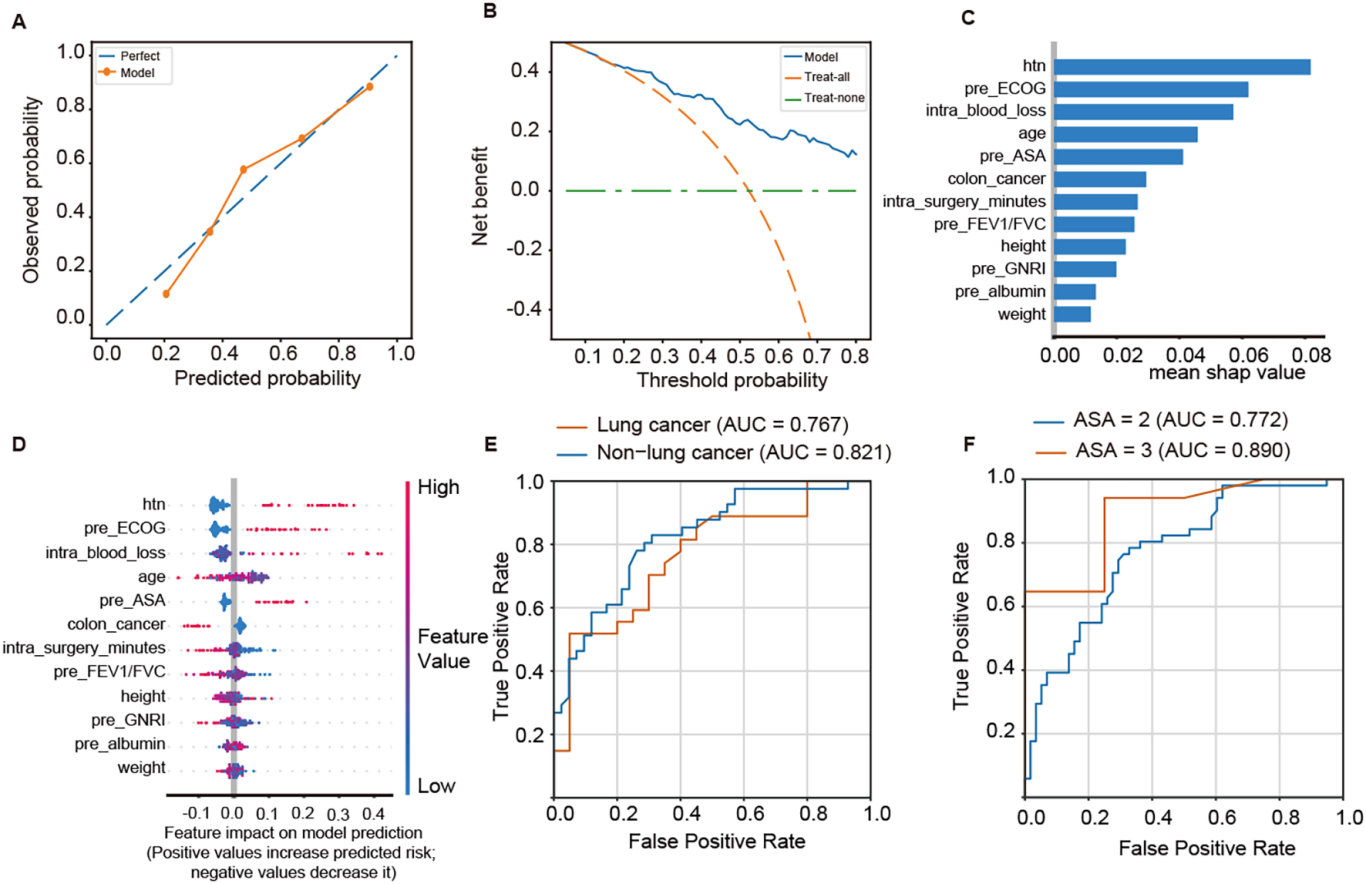
Interpretability and subgroup performance of SPC occurrence prediction model. **(A)** Calibration plot showing the agreement between predicted and observed probabilities of SPC occurrence. **(B)** Decision-curve analysis (DCA) demonstrating the net clinical benefit of the EXT model compared with “treat-all” and “treat-none” strategies. **(C)** Feature importance ranking derived from SHAP values. **(D)** SHAP summary plot illustrating the direction and magnitude of each feature’s contribution to the model output (positive SHAP values indicate higher risk of SPCs). **(E)** ROC curves for the lung cancer and non–lung cancer subgroups. **(F)** ROC curves for patients with ASA class II and class III.

Model calibration and clinical usefulness were evaluated using calibration and decision-curve analyses. The calibration curve demonstrated good agreement between the predicted and observed probabilities of SPC occurrence (**Figure 3A**). Decision-curve analysis indicated that the EXT model provided a higher net clinical benefit across a wide range of threshold probabilities compared with the “treat-all” or “treat-none” strategies (**Figure 3B**). Based on SHAP analysis, feature importance identified hypertension, preoperative ECOG score, intraoperative blood loss, and age as the strongest predictors of SPCs (**Figure 3C**). The SHAP analysis revealed that hypertension, preoperative ECOG score, intraoperative blood loss, age, and preoperative ASA score were among the strongest contributors to the model’s prediction of postoperative SPC (**Figure 3D**). Higher values of these variables were associated with an increased predicted risk of SPC. In contrast, a diagnosis of colon cancer, longer surgical duration, and better preoperative pulmonary function (FEV1/FVC%) were associated with negative SHAP values and suggested a protective effect against SPC occurrence. Subgroup analyses demonstrated stable discriminative performance across clinical subpopulations. The model achieved AUCs of 0.767 and 0.821 in the lung cancer and non–lung cancer groups, respectively (**Figure 3E**), and 0.772 and 0.890 in patients with ASA class II and class III, respectively (**Figure 3F**).

### 3.3 Model performance for 28-day mortality prediction

**Figure 4:**
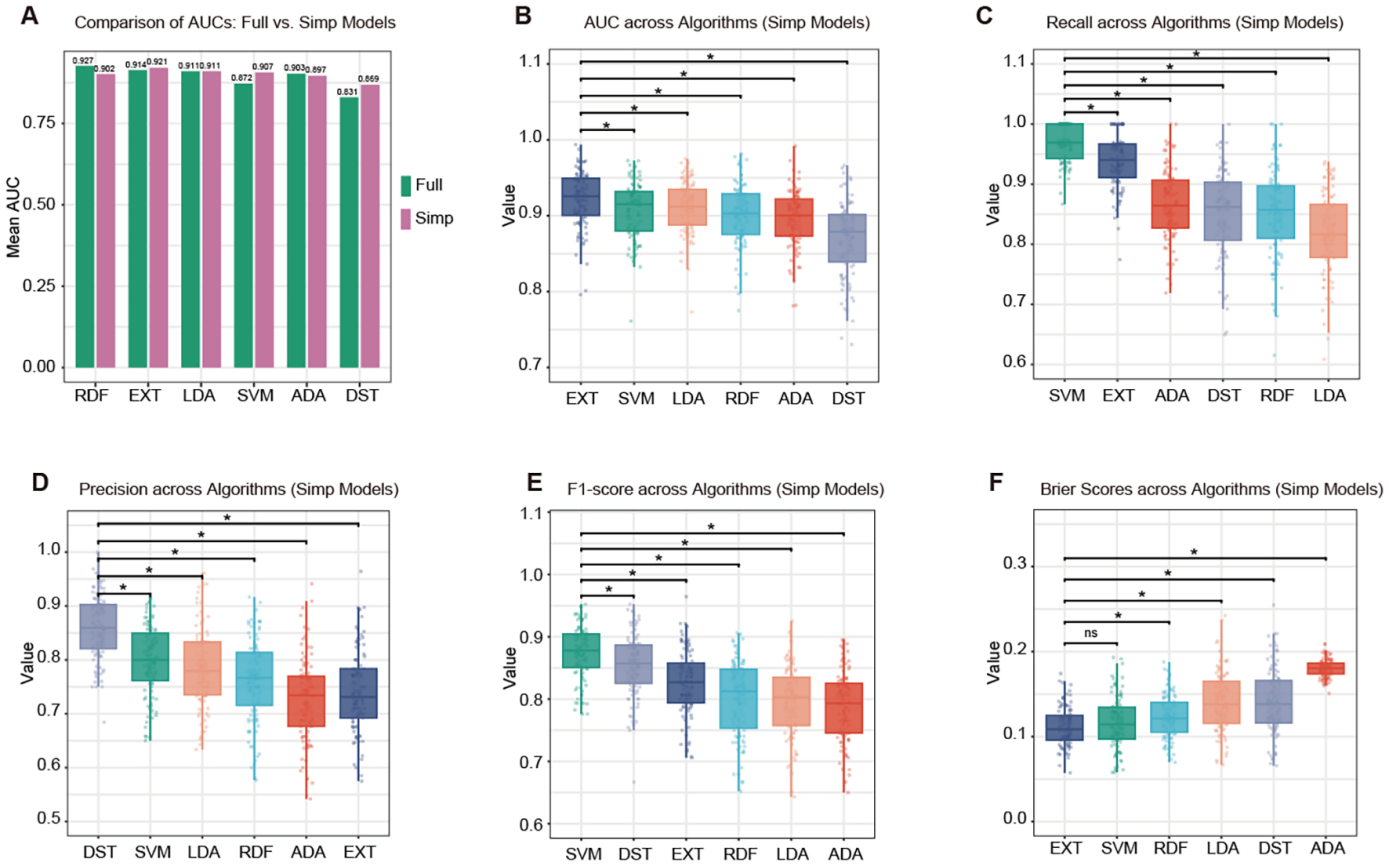
Model performance for predicting 28-day mortality. **(A)** Comparison of mean AUC values between models trained with all 56 available variables (Full) and those trained with the top 12 selected variables (Simp). Boxplots display the distributions of **(B)** AUC, **(C)** recall, **(D)** precision, **(E)** F1-score, and **(F)** Brier score derived from 100 bootstrap iterations. Horizontal lines denote statistically significant pairwise differences (*represents p-value<0.05; ns represents p-value>0.05).

Six machine learning classifiers were trained and evaluated using either all 56 available variables (model-full) or the top 12 most important predictors identified by random forest (model-simp) to predict 28-day mortality. As shown in **Figure 4A**, the RDF model achieved the highest AUC (0.927) among the full-feature models, while the EXT model obtained the highest AUC (0.921) among the simplified models. Notably, the AUC of EXT slightly increased from 0.914 in the full model to 0.921 in the simplified model. This result indicated that the reduced set of 12 key predictors preserved, and even improved, its discriminative ability. EXT exhibited the highest mean AUC of 0.921 (95% CI: 0.914–0.928; **Figure 4B**), SVM achieved the highest recall (0.970, 95% CI: 0.963–0.976; **Figure 4C**), DST demonstrated the best precision (0.860, 95% CI: 0.848–0.872; **Figure 4D**), SVM yielded the highest F1-score (0.874, 95% CI: 0.866–0.883; **Figure 4E**), and EXT produced the lowest Brier score (0.112, 95% CI: 0.107–0.116; **Figure 4F**). Across varying probability thresholds, the EXT model maintained high sensitivity at lower cut-offs and achieved progressively higher specificity at higher cut-offs. The model reached an optimal balance around thresholds of 0.5–0.6 (**Supplementary Table 7**), where sensitivity ranged from 0.83 to 0.93 and specificity from 0.77 to 0.87 for predicting 28-day mortality after SPC.

**Figure 5:**
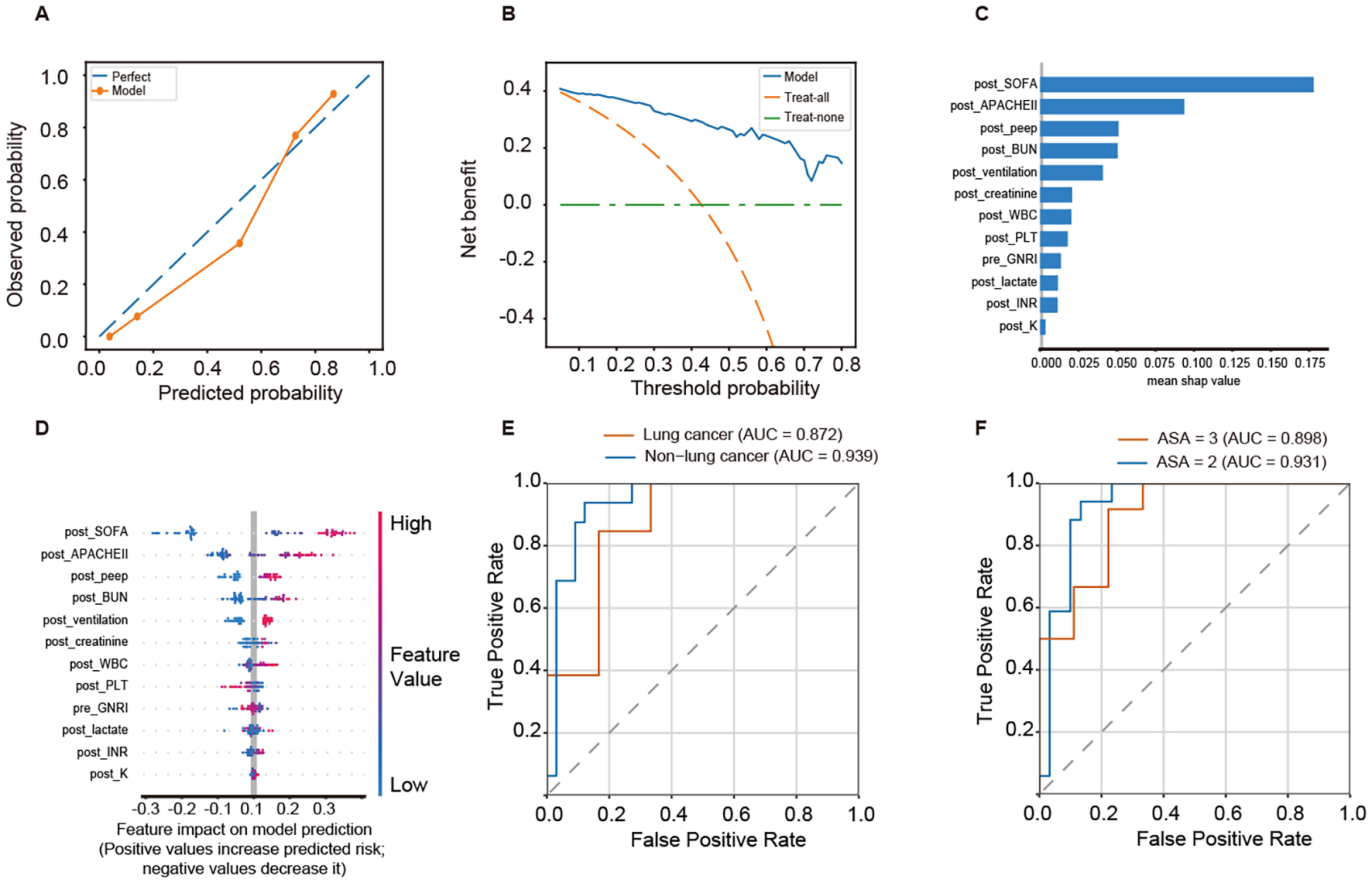
Interpretability and subgroup performance of 28-day mortality prediction model. **(A)** Calibration plot showing the agreement between predicted and observed probabilities of 28-day mortality. **(B)** Decision-curve analysis (DCA) demonstrating the net clinical benefit of the EXT model compared with “treat-all” and “treat-none” strategies. **(C)** Feature importance ranking derived from SHAP values. **(D)** SHAP summary plot illustrating the direction and magnitude of each feature’s contribution to the model output (positive SHAP values indicate higher risk of mortality). **(E)** ROC curves for the lung cancer and non–lung cancer subgroups. **(F)** ROC curves for patients with ASA class II and class III.

The calibration curve indicated excellent agreement between the predicted and observed probabilities of 28-day mortality (**Figure 5A**). Decision-curve analysis demonstrated that the EXT model provided greater net clinical benefit across a broad range of threshold probabilities compared with the “treat-all” and “treat-none” strategies (**Figure 5B**). According to SHAP analysis, postoperative SOFA score, postoperative APACHE II score, postoperative positive end-expiratory pressure (PEEP), postoperative blood urea nitrogen (BUN), and postoperative ventilation were identified as the strongest predictors of 28-day mortality (**Figure 5C**). The SHAP summary plot illustrated that higher postoperative SOFA and APACHE II scores, elevated PEEP and BUN levels, and mechanical ventilation were positively associated with increased mortality risk (**Figure 5D**). Subgroup analyses demonstrated robust discriminative performance across clinical subpopulations. The model achieved AUCs of 0.872 and 0.939 in the lung cancer and non–lung cancer groups, respectively (**Figure 5E**), and 0.931 and 0.898 in patients with ASA class II and class III, respectively (**Figure 5F**).

### 3.4 Model performance for 90-day mortality prediction

**Figure 6:**
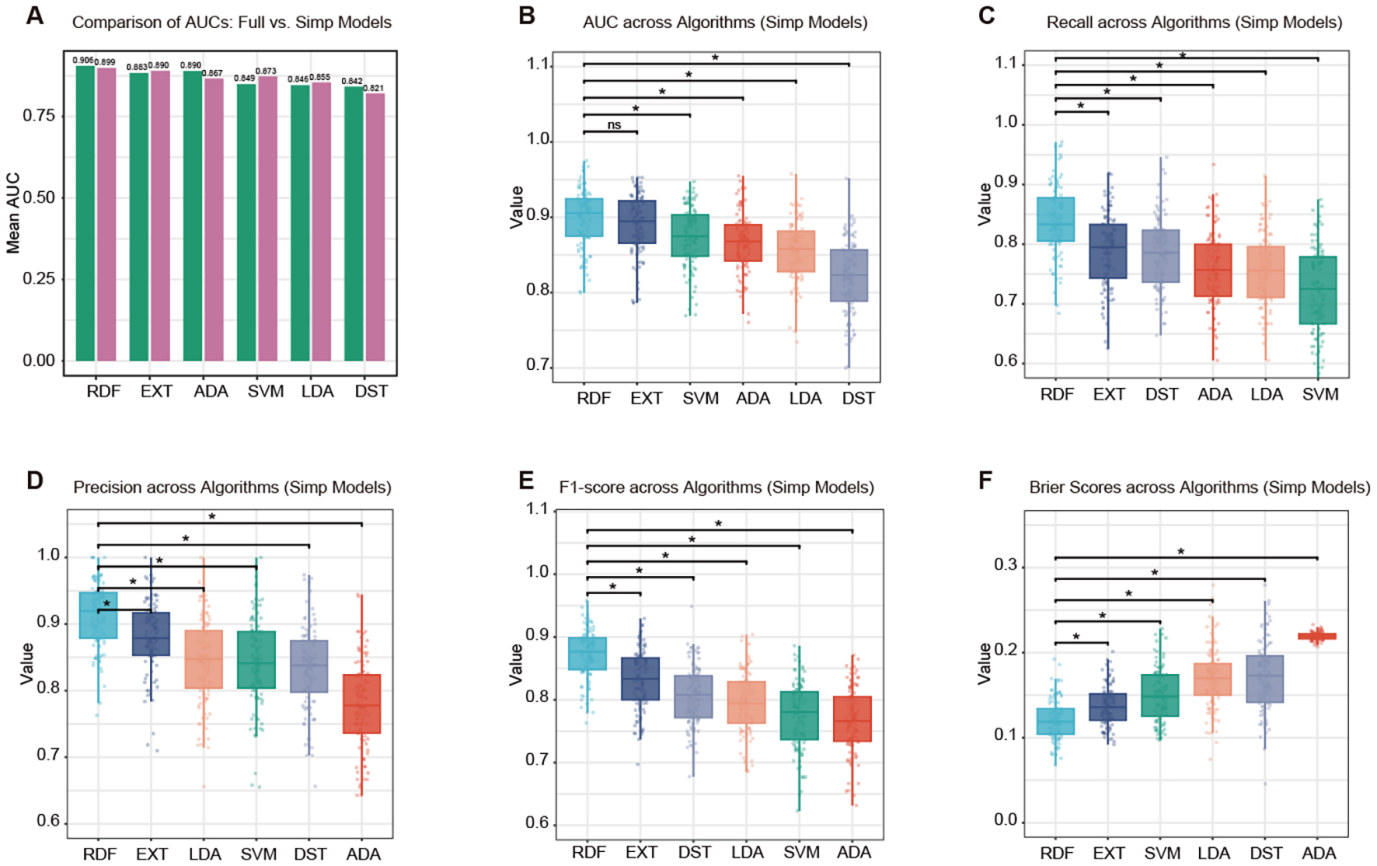
Model performance for predicting 90-day mortality. **(A)** Comparison of mean AUC values between models trained with all 56 available variables (Full) and those trained with the top 12 selected variables (Simp). Boxplots display the distributions of **(B)** AUC, **(C)** recall, **(D)** precision, **(E)** F1-score, and **(F)** Brier score derived from 100 bootstrap iterations. Horizontal lines denote statistically significant pairwise differences (*represents p-value<0.05; ns represents p-value>0.05).

Six machine learning classifiers were developed to predict 90-day mortality using either all 56 available variables (model-full) or the top 12 predictors selected according to their feature importance scores (model-simp). As illustrated in **Figure 6A**, the RDF model achieved the highest AUC (0.906) among the full-feature models and the highest AUC (0.899) among the simplified models. The small performance gap between model-full and model-simp indicates that the simplified model maintained strong predictive ability despite the reduction in input variables. Among the simplified models, RDF consistently demonstrated superior performance. RDF achieved the highest mean AUC of 0.899 (95% CI: 0.891–0.907; **Figure 6B**), the greatest recall (0.840, 95% CI: 0.828–0.852; **Figure 6C**), the highest precision (0.912, 95% CI: 0.903–0.922; **Figure 6D**), the best F1-score (0.873, 95% CI: 0.865–0.881; **Figure 6E**), and the lowest Brier score (0.121, 95% CI: 0.116–0.126; **Figure 6F**). Across different probability thresholds, the RDF model showed high sensitivity at lower cut-offs and progressively improved specificity at higher cut-offs (**Supplementary Table 8**). The best balance between sensitivity and specificity was observed around thresholds of 0.45–0.60, where sensitivity ranged from 0.73 to 0.84 and specificity from 0.87 to 0.94 for predicting 90-day mortality after SPC.

**Figure 7:**
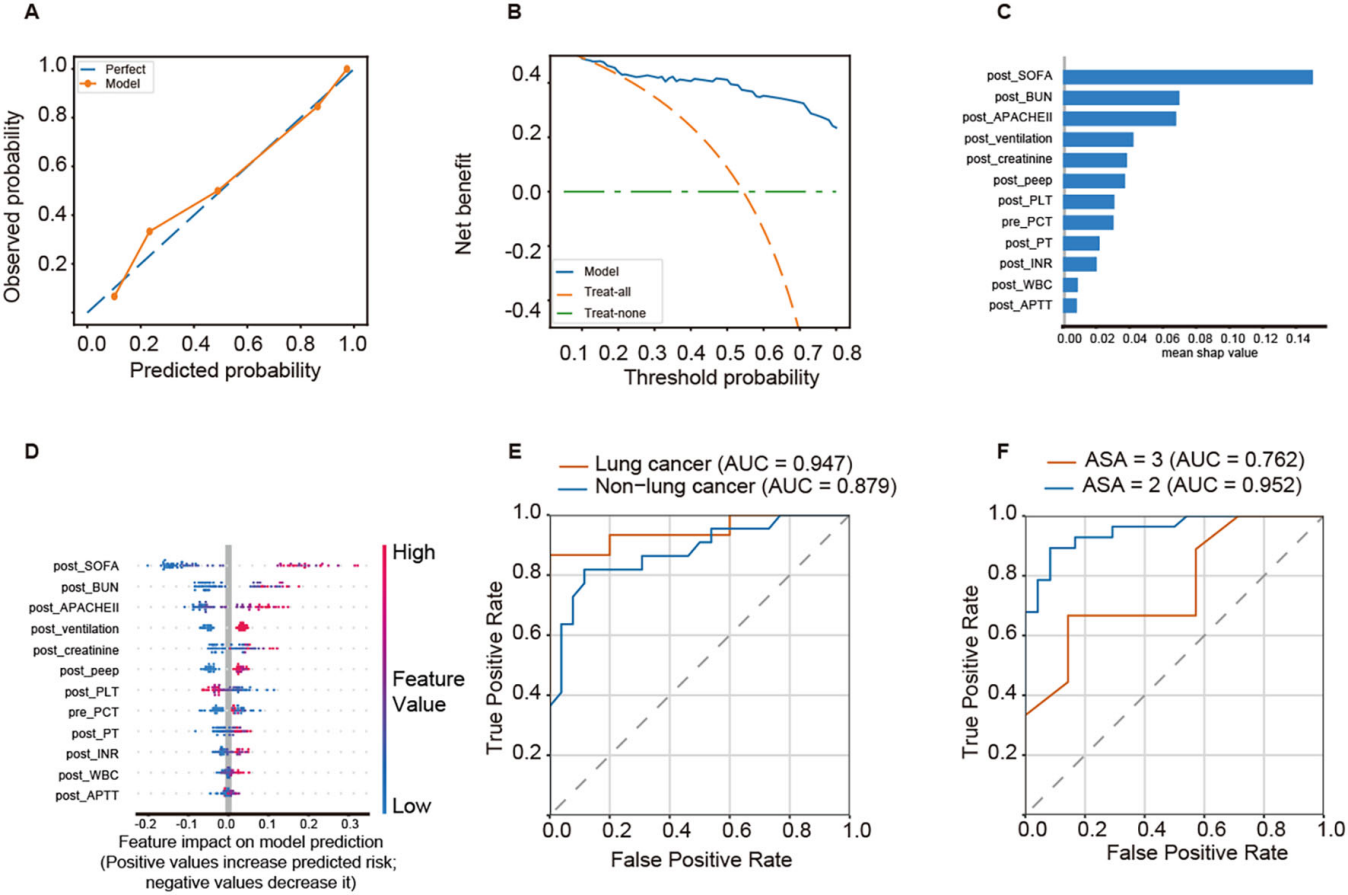
Interpretability and subgroup performance of 90-day mortality prediction model. **(A)** Calibration plot showing the agreement between predicted and observed probabilities of 90-day mortality. **(B)** DCA demonstrating the net clinical benefit of the RDF model compared with “treat-all” and “treat-none” strategies. **(C)** Feature importance ranking derived from SHAP values. **(D)** SHAP summary plot illustrating the direction and magnitude of each feature’s contribution to the model output (positive SHAP values indicate higher risk of mortality). **(E)** ROC curves for the lung cancer and non–lung cancer subgroups. **(F)** ROC curves for patients with ASA class II and class III.

The calibration plot revealed close alignment between predicted and observed mortality probabilities of 90-day mortality (**Figure 7A**). Decision-curve analysis (**Figure 7B**) demonstrated that the RDF model achieved higher net clinical benefit across a broad range of threshold probabilities compared with the “treat-all” and “treat-none” strategies. SHAP-based interpretability analysis (**Figures 7C, D**) identified postoperative SOFA score, postoperative BUN, postoperative APACHE II score, postoperative ventilation, and postoperative creatinine as the top predictors of 90-day mortality. As shown in the SHAP summary plot, elevated postoperative SOFA, APACHE II, BUN, and creatinine levels, as well as the need for mechanical ventilation, were strongly associated with increased mortality risk. Subgroup analyses demonstrated robust model performance across clinical categories. The model achieved AUCs of 0.947 and 0.879 in the lung cancer and non–lung cancer groups, respectively (**Figure 7E**), and AUCs of 0.952 and 0.762 in patients with ASA class II and class III, respectively (**Figure 7F**). These findings suggest that the model retained excellent discriminative ability and clinical generalizability across subpopulations.

### 3.5 Web-based deployment

**Figure 8:**
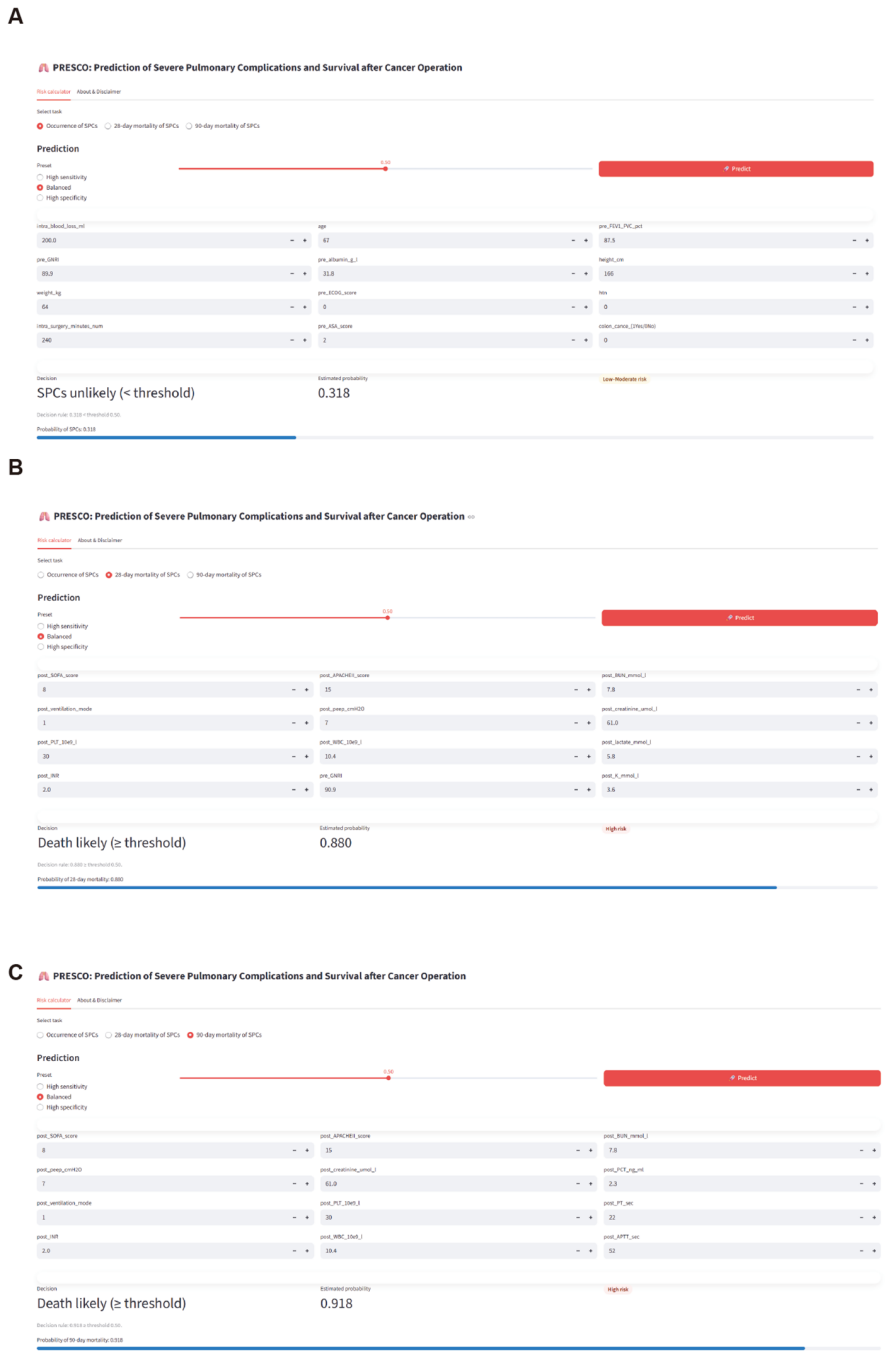
Web-based prediction applications for postoperative respiratory failure, pulmonary embolism, and mortality. PRESCO (https://presco.streamlit.app/), an online tool, was developed to enable real-time prediction of severe pulmonary complications and survival outcomes. **(A)** Occurrence prediction application for secondary pulmonary complications (SPCs). **(B)** Prediction application for 28-day mortality. **(C)** Prediction application for 90-day mortality.

The models with the highest AUC values were integrated into an online tool, PRESCO (https://presco.streamlit.app/), which comprises three applications for predicting the occurrence of SPC (**Figure 8A**), 28-day mortality of SPC (**Figure 8B**), and 90-day mortality of SPC (**Figure 8C**). Each application enables clinicians to input 12 selected features and instantly generates predictions of occurrence risk or survival probability. The outputs include both categorical classifications and predicted probabilities.

As an illustration, we randomly selected a patient (ID1954417) to demonstrate the use of the occurrence prediction application (**Figure 5A**). The patient belongs to the normal group, with intraoperative blood loss of 200 mL and age 67. The preoperative FEV1/FVC ratio is 87.5%, pre-GNRI is 89.9, and pre-albumin is 31.8 g/L. The patient’s height is 166 cm and weight is 64 kg. The preoperative ECOG score is 0, with no hypertension. The surgery lasted 240 minutes, the pre-ASA score is 2, and the patient does not have colon cancer. The model estimated a probability of 0.318 for the occurrence of SPCs, which was below the decision threshold of 0.50. Accordingly, the prediction result was “SPCs unlikely (< threshold)”, indicating that this patient was at low risk of developing postoperative SPCs. This example demonstrated the practical application of the prediction tool for individual risk assessment.

## 4 Discussion

We developed and validated machine learning models to predict the occurrence and survival outcome of SPCs in cancer patients. These models rely on only 12 easily extractable variables. It demonstrated excellent discriminatory performance in predicting postoperative SPC with AUC of 0.813. It also could be used for survival prediction for SPCs patients in 28 and 90 days, with an AUC of 0.921 and 0.899. We developed an online web server to facilitate the implementation and generate the prediction result.

One of the key strengths of this study is its ability to balance high predictive accuracy (AUC > 0.80–0.90) with practical clinical usability through a readily accessible online platform. The trained machine learning models were translated into intuitive web-based applications that allow clinicians to obtain patient-specific risk predictions and model explanations in real time. This user-friendly interface enables rapid bedside assessment and supports early, individualized interventions such as intensified monitoring, prophylactic anticoagulation, and timely escalation of respiratory support. Another important strength of our study is that it provides a comprehensive decision-support toolkit rather than focusing on a single outcome. In addition to predicting the occurrence of SPCs, our framework also incorporates models for survival prediction. Previous studies have typically targeted only a single endpoint. For example, one study developed a machine learning–based risk prediction model for postoperative deep vein thrombosis in cancer patients (12), while another proposed an XGBoost model to predict in-hospital mortality in cancer patients with acute pulmonary embolism (13). Relying on multiple separate tools is neither efficient nor convenient in routine clinical practice. In contrast, our integrated platform allows clinicians to evaluate SPC occurrence together with short- and long-term prognosis within one system, which results in a more comprehensive understanding of perioperative risk. By consolidating multiple outcomes, our tool has the potential to better support individualized treatment planning and optimize perioperative resource allocation.

Previous studies have indicated that postoperative SPC arises from a multifactorial interplay of patient-related factors (e.g., advanced age, comorbidities, and pulmonary disease), surgical (e.g., site, duration, urgency, and approach), and anesthesia-related factors (e.g., technique and ventilation management) (14). Consistent with these findings, our analysis identified hypertension, preoperative ECOG score, intraoperative blood loss, age, and preoperative ASA score as significant positive predictors of SPC. Notably, patients with high intraoperative blood loss have been reported to experience a significantly higher incidence of SPC than those with low blood loss (15), supporting our observation that both preoperative comorbidities and intraoperative events contribute substantially to the occurrence of SPC.

Among patients who developed SPC, higher postoperative SOFA and APACHE II scores, elevated PEEP and BUN levels, and the need for mechanical ventilation were strongly associated with increased mortality risk. These findings align with previous studies, which demonstrate that elevated SOFA scores are associated with higher risks of venous thromboembolism and mortality, and that SOFA, APACHE II, and BUN are independent predictors of mortality (16, 17). Collectively, our results suggest that integrating routinely available clinical parameters across preoperative, intraoperative, and postoperative phases into predictive models may facilitate early risk stratification, timely intervention, and ultimately improved patient outcomes.

Nevertheless, several limitations must be acknowledged. First, this study was conducted retrospectively in a single cancer center, which may introduce institutional and regional bias and limit the generalizability of the findings. Therefore, external validation using multicenter cohorts and prospective studies is essential to confirm the robustness, reproducibility, and clinical applicability of the PRESCO model in diverse healthcare settings. Second, while our feature set was comprehensive, some potentially important variables, such as detailed intraoperative ventilation parameters or postoperative rehabilitation measures, were unavailable. Finally, although we deployed the models online, prospective evaluation of their clinical utility and cost-effectiveness in routine care is still needed.

## 5 Conclusion

In conclusion, we developed and validated machine learning models for predicting the occurrence of postoperative pulmonary complications in surgical cancer patients, as well as 28-day and 90-day mortality among affected patients. Deployment as browser-based applications ensures accessibility and practicality for users. These models have the potential to support early identification of high-risk patients, guide perioperative management strategies, and ultimately improve outcomes in surgical oncology.

## Data Availability

The original contributions presented in the study are included in the article/**Supplementary Material**. Further inquiries can be directed to the corresponding author.
