# Supplementary material for: PRESCO: an online tool for predicting severe pulmonary complications and survival after cancer surgery
Source: Front Oncol. 2026 Jan 7;15:1705181. doi: 10.3389/fonc.2025.1705181 (PMC12819265; doi:10.3389/fonc.2025.1705181)
Supplement: Supplementary file 1 [file Table1.docx]

| Variable | Missing Number | Missing Percent |
| --- | --- | --- |
| T stage | 163 | 37.56 |
| N stage | 156 | 35.94 |
| M stage | 142 | 32.72 |
| Pre FEV1 FVC | 82 | 18.89 |
| Intra surgery minutes | 10 | 2.30 |
| Intra blood loss | 14 | 3.23 |

**SupTable 1. Summary of missing values for each variable in the dataset used to predict SPC occurrence.** Preoperative forced expiratory volume in 1 second/forced vital capacity ratio (pre FEV1 FVC); duration of surgery (Intra surgery minutes), intraoperative blood loss (Intra blood loss).
