# Supplementary material for: PRESCO: an online tool for predicting severe pulmonary complications and survival after cancer surgery
Source: Front Oncol. 2026 Jan 7;15:1705181. doi: 10.3389/fonc.2025.1705181 (PMC12819265; doi:10.3389/fonc.2025.1705181)
Supplement: Supplementary file 2 [file Table2.docx]

| Variable | Missing Number | Missing Percent |
| --- | --- | --- |
| T stage | 84 | 37.00 |
| N stage | 81 | 35.68 |
| M stage | 73 | 32.16 |
| Pre FEV1/FVC | 48 | 21.15 |
| Intra surgery minutes | 10 | 4.41 |
| Intra blood loss | 14 | 6.17 |
| Post paco2 | 1 | 0.44 |
| Post fio2 | 29 | 12.78 |
| Post peep | 3 | 1.32 |
| Post MAP | 3 | 1.32 |
| Post SBP | 3 | 1.32 |
| Post DBP | 3 | 1.32 |
| Post PCT | 15 | 6.61 |
| Post CRP | 75 | 33.04 |
| Post albumin | 2 | 0.88 |
| Post HCO3 | 1 | 0.44 |
| Post arrhythmia | 1 | 0.44 |

**SupTable 2. Summary of missing values by variable in the dataset used for predicting 28-day and 90-day mortality outcomes.** Preoperative forced expiratory volume in 1 second/forced vital capacity ratio (FEV1/FVC), duration of surgery (Intra surgery minutes), intraoperative blood loss (Intra blood loss); Postoperative arterial partial pressure of carbon dioxide (post paco2), fraction of inspired oxygen (post fio2), positive end-expiratory pressure (post peep); Postoperative mean arterial pressure (Post MAP), systolic blood pressure (Post SBP), diastolic blood pressure (Post DBP), procalcitonin (Post PCT), C-reactive protein (Post CRP), bicarbonate (Post HCO₃).
