# Supplementary material for: PRESCO: an online tool for predicting severe pulmonary complications and survival after cancer surgery
Source: Front Oncol. 2026 Jan 7;15:1705181. doi: 10.3389/fonc.2025.1705181 (PMC12819265; doi:10.3389/fonc.2025.1705181)
Supplement: Supplementary file 3 [file Table3.docx]

|  | **Training** | **Testing** | **p-value** |
| --- | --- | --- | --- |
|  | (n=304) | (n=130) |  |
| age | 60.4 (12.6) | 62.4 (10.4) | 0.083 |
| sex: |  |  | 0.767 |
| Female | 142 (46.7%) | 58 (44.6%) |  |
| Male | 162 (53.3%) | 72 (55.4%) |  |
| Height (cm) | 161 (7.18) | 161 (6.75) | 0.578 |
| Weight (kg) | 60.2 (10.9) | 60.1 (9.75) | 0.921 |
| Tumor type: |  |  | 0.495 |
| Cervical cancer | 34 (11.2%) | 12 (9.23%) |  |
| Colon cancer | 43 (14.1%) | 16 (12.3%) |  |
| Esophageal cancer | 25 (8.22%) | 16 (12.3%) |  |
| Lung cancer | 91 (29.9%) | 47 (36.2%) |  |
| others | 77 (25.3%) | 27 (20.8%) |  |
| Ovarian cancer | 34 (11.2%) | 12 (9.23%) |  |
| T stage: |  |  | 0.832 |
| T1 | 36 (11.8%) | 19 (14.6%) |  |
| T2 | 175 (57.6%) | 72 (55.4%) |  |
| T3 | 53 (17.4%) | 24 (18.5%) |  |
| T4 | 40 (13.2%) | 15 (11.5%) |  |
| N stage: |  |  | 0.791 |
| N0 | 208 (68.4%) | 83 (63.8%) |  |
| N1 | 48 (15.8%) | 25 (19.2%) |  |
| N2 | 36 (11.8%) | 17 (13.1%) |  |
| N3 | 12 (3.95%) | 5 (3.85%) |  |
| M stage: |  |  | 0.643 |
| M0 | 264 (86.8%) | 110 (84.6%) |  |
| M1 | 40 (13.2%) | 20 (15.4%) |  |
| htn: |  |  | 0.885 |
| No | 249 (81.9%) | 105 (80.8%) |  |
| Yes | 55 (18.1%) | 25 (19.2%) |  |
| dm: |  |  | 0.938 |
| No | 283 (93.1%) | 122 (93.8%) |  |
| Yes | 21 (6.91%) | 8 (6.15%) |  |
| cad: |  |  | 0.119 |
| No | 293 (96.4%) | 129 (99.2%) |  |
| Yes | 11 (3.62%) | 1 (0.77%) |  |
| stroke: |  |  | 0.638 |
| No | 301 (99.0%) | 128 (98.5%) |  |
| Yes | 3 (0.99%) | 2 (1.54%) |  |
| Pre ASA score | 2.15 (0.37) | 2.16 (0.37) | 0.858 |
| Pre ECOG score | 0.28 (0.57) | 0.22 (0.47) | 0.258 |
| Pre GNRI | 104 (11.4) | 104 (11.5) | 0.979 |
| Pre albumin | 39.7 (5.07) | 39.5 (4.54) | 0.714 |
| Pre FEV1/FVC | 87.0 (5.53) | 87.5 (5.92) | 0.414 |
| Intra surgery minutes | 213 (94.5) | 203 (83.0) | 0.258 |
| Intra blood loss | 301 (440) | 250 (201) | 0.098 |
| group: |  |  | 1.000 |
| Normal | 145 (47.7%) | 62 (47.7%) |  |
| SPC | 159 (52.3%) | 68 (52.3%) |  |

**supTable 3. Clinical and laboratory variables included in the SPC occurrence prediction model**. This table summarizes the demographic, oncological, comorbidity, perioperative, variables used for model construction. Variables included: age; sex (female or male); height (cm); weight (kg); tumor type (cervical, colon, esophageal, lung, ovarian cancer, or others); TNM stage (T, N, M); comorbidities [hypertension (htn), diabetes mellitus (dm), coronary artery disease (cad), and stroke]; preoperative assessments [American Society of Anesthesiologists (ASA) score, Eastern Cooperative Oncology Group (ECOG) performance status, Geriatric Nutritional Risk Index (GNRI), preoperative serum albumin, forced expiratory volume in 1 second/forced vital capacity ratio (FEV1/FVC, %)], perioperative factors [duration of surgery (minutes), intraoperative blood loss (mL)].
