# Supplementary material for: PRESCO: an online tool for predicting severe pulmonary complications and survival after cancer surgery
Source: Front Oncol. 2026 Jan 7;15:1705181. doi: 10.3389/fonc.2025.1705181 (PMC12819265; doi:10.3389/fonc.2025.1705181)
Supplement: Supplementary file 5 [file Table5.docx]

|  | **Training** | **Testing** | **p-value** |
| --- | --- | --- | --- |
|  | (n=159) | (n=68) |  |
| group: |  |  | 0.941 |
| alive | 75 (47.2%) | 31 (45.6%) |  |
| dead | 84 (52.8%) | 37 (54.4%) |  |
| age | 61.9 (11.3) | 61.0 (10.9) | 0.574 |
| sex: |  |  | 0.759 |
| Female | 73 (45.9%) | 29 (42.6%) |  |
| Male | 86 (54.1%) | 39 (57.4%) |  |
| Weight (kg) | 59.3 (10.2) | 62.2 (11.1) | 0.070 |
| Height (cm) | 161 (7.66) | 162 (7.41) | 0.449 |
| Tumor type: |  |  | 0.928 |
| Cervical cancer | 9 (5.66%) | 6 (8.82%) |  |
| Colon cancer | 12 (7.55%) | 4 (5.88%) |  |
| Endometrial cancer | 8 (5.03%) | 3 (4.41%) |  |
| Esophageal cancer | 12 (7.55%) | 7 (10.3%) |  |
| Lung cancer | 45 (28.3%) | 20 (29.4%) |  |
| others | 59 (37.1%) | 24 (35.3%) |  |
| Ovarian cancer | 14 (8.81%) | 4 (5.88%) |  |
| T stage: |  |  | 0.028 |
| T1 | 13 (8.18%) | 15 (22.1%) |  |
| T2 | 91 (57.2%) | 36 (52.9%) |  |
| T3 | 32 (20.1%) | 9 (13.2%) |  |
| T4 | 23 (14.5%) | 8 (11.8%) |  |
| N stage: |  |  | 0.546 |
| N0 | 106 (66.7%) | 46 (67.6%) |  |
| N1 | 24 (15.1%) | 13 (19.1%) |  |
| N2 | 20 (12.6%) | 8 (11.8%) |  |
| N3 | 9 (5.66%) | 1 (1.47%) |  |
| M stage: |  |  | 0.971 |
| M0 | 136 (85.5%) | 59 (86.8%) |  |
| M1 | 23 (14.5%) | 9 (13.2%) |  |
| htn: |  |  | 0.564 |
| No | 113 (71.1%) | 45 (66.2%) |  |
| Yes | 46 (28.9%) | 23 (33.8%) |  |
| dm: |  |  | 0.545 |
| No | 146 (91.8%) | 60 (88.2%) |  |
| Yes | 13 (8.18%) | 8 (11.8%) |  |
| cad: |  |  | 0.728 |
| No | 152 (95.6%) | 66 (97.1%) |  |
| Yes | 7 (4.40%) | 2 (2.94%) |  |
| stroke: |  |  | 1.000 |
| No | 157 (98.7%) | 68 (100%) |  |
| Yes | 2 (1.26%) | 0 (0.00%) |  |
| Pre ASA | 2.26 (0.45) | 2.24 (0.43) | 0.721 |
| Pre albumin | 39.7 (5.34) | 39.7 (4.18) | 1.000 |
| Pre GNRI | 104 (11.2) | 105 (10.6) | 0.364 |
| Pre FEV1/FVC t | 87.0 (6.06) | 86.7 (6.18) | 0.676 |
| Pre ECOG | 0.42 (0.62) | 0.35 (0.69) | 0.521 |
| Intra surgery minutes | 208 (95.7) | 203 (120) | 0.761 |
| Intra blood loss | 382 (606) | 246 (181) | 0.011 |
| Post ventilation time | 6.99 (19.3) | 6.57 (17.2) | 0.871 |
| Post pao2 | 68.1 (20.6) | 75.9 (32.4) | 0.072 |
| Post paco2 | 38.5 (9.57) | 39.9 (14.1) | 0.440 |
| Post ph | 7.39 (0.11) | 7.37 (0.13) | 0.168 |
| Post fio2 | 0.73 (0.24) | 0.76 (0.23) | 0.321 |
| Post pao2/fio2 | 115 (59.1) | 118 (69.8) | 0.770 |
| Post ventilation |  |  | 0.897 |
| No | 67 (42.1%) | 30 (44.1%) |  |
| Yes | 92 (57.9%) | 38 (55.9%) |  |
| Post peep | 3.82 (3.63) | 3.44 (3.53) | 0.460 |
| Post SOFA | 5.82 (4.43) | 5.69 (4.05) | 0.834 |
| Post APACHEII | 15.4 (7.37) | 15.3 (6.97) | 0.917 |
| Post MAP | 90.4 (19.2) | 91.9 (21.8) | 0.627 |
| Post SBP | 122 (28.1) | 127 (32.6) | 0.263 |
| Post DBP | 74.9 (16.3) | 74.4 (18.0) | 0.868 |
| Post HR | 108 (24.6) | 108 (25.2) | 0.970 |
| Post lactate | 2.98 (3.22) | 3.58 (3.79) | 0.256 |
| Post WBC | 15.9 (16.7) | 15.8 (8.20) | 0.983 |
| Post PCT | 7.42 (19.6) | 5.47 (12.9) | 0.380 |
| Post CRP | 117 (70.6) | 94.8 (69.0) | 0.031 |
| Post Hb | 93.4 (19.2) | 94.8 (19.4) | 0.612 |
| Post PLT | 215 (137) | 225 (125) | 0.598 |
| Post creatinine | 101 (95.7) | 107 (97.6) | 0.668 |
| Post BUN | 9.90 (8.49) | 10.3 (8.84) | 0.777 |
| Post albumin | 29.8 (4.68) | 29.4 (4.94) | 0.618 |
| Post PT | 14.7 (4.35) | 14.6 (3.81) | 0.934 |
| Post INR | 1.30 (0.42) | 1.30 (0.35) | 0.977 |
| Post APTT | 33.0 (10.1) | 32.6 (8.65) | 0.751 |
| Post glucose | 10.3 (4.40) | 10.2 (3.58) | 0.884 |
| Post HCO3 | 23.7 (4.89) | 21.9 (4.53) | 0.011 |
| Post Na | 138 (6.35) | 139 (5.07) | 0.248 |
| Post K | 3.82 (0.64) | 3.86 (0.60) | 0.601 |
| Post infection |  |  | 0.845 |
| No | 50 (31.4%) | 23 (33.8%) |  |
| Yes | 109 (68.6%) | 45 (66.2%) |  |
| Post delirium |  |  | 0.907 |
| No | 129 (81.1%) | 54 (79.4%) |  |
| Yes | 30 (18.9%) | 14 (20.6%) |  |
| Post aki |  |  | 1.000 |
| No | 122 (76.7%) | 52 (76.5%) |  |
| Yes | 37 (23.3%) | 16 (23.5%) |  |
| Post arrhythmia |  |  | 0.854 |
| No | 130 (81.8%) | 57 (83.8%) |  |
| Yes | 29 (18.2%) | 11 (16.2%) |  |
| Post dvt |  |  | 0.763 |
| No | 117 (73.6%) | 48 (70.6%) |  |
| Yes | 42 (26.4%) | 20 (29.4%) |  |
| Post sedative |  |  | 0.606 |
| No | 22 (13.8%) | 7 (10.3%) |  |
| Yes | 137 (86.2%) | 61 (89.7%) |  |
| disease: |  |  | 1.000 |
| PE | 85 (53.5%) | 37 (54.4%) |  |
| PRF | 74 (46.5%) | 31 (45.6%) |  |

**supTable 5. Clinical, perioperative, and postoperative variables for 90-days survival prediction in patients with secondary pulmonary complications (SPCs).** Variables comprised: Outcome: group (alive, dead); Demographics: age, sex (female, male), weight (kg), height (cm); Tumor characteristics: tumor type, TNM stage [T stage (T1–T4), N stage (N0–N3), M stage (M0–M1)]; Comorbidities: hypertension (htn: Yes/No), diabetes mellitus (dm: Yes/No), coronary artery disease (cad: Yes/No), stroke (Yes/No); Preoperative evaluation and nutritional/functional status: American Society of Anesthesiologists (ASA) score, serum albumin (g/L), Geriatric Nutritional Risk Index (GNRI), forced expiratory volume in 1 second/forced vital capacity ratio (FEV1/FVC, %), Eastern Cooperative Oncology Group (ECOG) performance status; Perioperative factors: duration of surgery (minutes), intraoperative blood loss (mL); Postoperative respiratory support and gas exchange: mechanical ventilation time (hours), arterial partial pressure of oxygen (PaO₂, mmHg), arterial partial pressure of carbon dioxide (PaCO₂, mmHg), pH, fraction of inspired oxygen (FiO₂, %), oxygenation index (PaO₂/FiO₂), ventilation mode (Yes/No), positive end-expiratory pressure (PEEP, cmH₂O); Postoperative severity scores and vital signs: Sequential Organ Failure Assessment (SOFA) score, Acute Physiology and Chronic Health Evaluation II (APACHE II) score, mean arterial pressure (MAP, mmHg), systolic blood pressure (SBP, mmHg), diastolic blood pressure (DBP, mmHg), heart rate (HR, bpm), blood lactate (mmol/L); Postoperative laboratory findings: white blood cell count (WBC, ×10⁹/L), procalcitonin (PCT, ng/mL), C-reactive protein (CRP, mg/L), hemoglobin (Hb, g/L), platelet count (PLT, ×10⁹/L), serum creatinine (µmol/L), blood urea nitrogen (BUN, mmol/L), serum albumin (g/L), prothrombin time (PT, seconds), international normalized ratio (INR), activated partial thromboplastin time (APTT, seconds), blood glucose (mmol/L), bicarbonate (HCO₃⁻, mmol/L), sodium (Na⁺, mmol/L), potassium (K⁺, mmol/L); Postoperative complications and events: infection (Yes/No), delirium (Yes/No), acute kidney injury (AKI, Yes/No), arrhythmia (Yes/No), deep vein thrombosis (DVT, Yes/No), use of sedative medications (Yes/No); Disease type: pulmonary embolism (PE) or postoperative respiratory failure (PRF).
