# Supplementary material for: PRESCO: an online tool for predicting severe pulmonary complications and survival after cancer surgery
Source: Front Oncol. 2026 Jan 7;15:1705181. doi: 10.3389/fonc.2025.1705181 (PMC12819265; doi:10.3389/fonc.2025.1705181)
Supplement: Supplementary file 6 [file Table6.docx]

| **threshold** | **sensitivity** | **specificity** | **PPV** | **NPV** |
| --- | --- | --- | --- | --- |
| 0.05 | 1.000 | 0.000 | 0.523 | - |
| 0.10 | 1.000 | 0.016 | 0.527 | 1.000 |
| 0.15 | 0.985 | 0.081 | 0.540 | 0.833 |
| 0.20 | 0.985 | 0.145 | 0.558 | 0.900 |
| 0.25 | 0.985 | 0.290 | 0.604 | 0.947 |
| 0.30 | 0.941 | 0.371 | 0.621 | 0.852 |
| 0.35 | 0.868 | 0.484 | 0.648 | 0.769 |
| 0.40 | 0.853 | 0.613 | 0.707 | 0.792 |
| 0.45 | 0.750 | 0.710 | 0.739 | 0.721 |
| 0.50 | 0.647 | 0.758 | 0.746 | 0.662 |
| 0.55 | 0.574 | 0.839 | 0.796 | 0.642 |
| 0.60 | 0.515 | 0.871 | 0.814 | 0.621 |
| 0.65 | 0.500 | 0.919 | 0.872 | 0.626 |
| 0.70 | 0.456 | 0.935 | 0.886 | 0.611 |
| 0.75 | 0.441 | 0.935 | 0.882 | 0.604 |
| 0.80 | 0.412 | 0.952 | 0.903 | 0.596 |
| 0.85 | 0.279 | 0.968 | 0.905 | 0.550 |
| 0.90 | 0.206 | 0.984 | 0.933 | 0.530 |
| 0.95 | 0.059 | 1.000 | 1.000 | 0.492 |

supTable 6. Threshold‐dependent diagnostic performance of the simplified EXT model for predicting severe pulmonary complications (SPCs) in the test cohort, showing sensitivity, specificity, positive predictive value (PPV), and negative predictive value (NPV) at different predicted probability cut-offs.
