# Supplementary material for: PRESCO: an online tool for predicting severe pulmonary complications and survival after cancer surgery
Source: Front Oncol. 2026 Jan 7;15:1705181. doi: 10.3389/fonc.2025.1705181 (PMC12819265; doi:10.3389/fonc.2025.1705181)
Supplement: Supplementary file 7 [file Table7.docx]

| **threshold** | **sensitivity** | **specificity** | **PPV** | **NPV** |
| --- | --- | --- | --- | --- |
| 0.05 | 1.000 | 0.385 | 0.547 | 1.000 |
| 0.10 | 1.000 | 0.436 | 0.569 | 1.000 |
| 0.15 | 1.000 | 0.615 | 0.659 | 1.000 |
| 0.20 | 1.000 | 0.641 | 0.674 | 1.000 |
| 0.25 | 1.000 | 0.641 | 0.674 | 1.000 |
| 0.30 | 0.966 | 0.667 | 0.683 | 0.963 |
| 0.35 | 0.966 | 0.692 | 0.700 | 0.964 |
| 0.40 | 0.966 | 0.692 | 0.700 | 0.964 |
| 0.45 | 0.931 | 0.744 | 0.730 | 0.935 |
| 0.50 | 0.931 | 0.769 | 0.750 | 0.938 |
| 0.55 | 0.897 | 0.821 | 0.788 | 0.914 |
| 0.60 | 0.828 | 0.872 | 0.828 | 0.872 |
| 0.65 | 0.828 | 0.872 | 0.828 | 0.872 |
| 0.70 | 0.690 | 0.897 | 0.833 | 0.795 |
| 0.75 | 0.552 | 0.949 | 0.889 | 0.740 |
| 0.80 | 0.483 | 0.974 | 0.933 | 0.717 |
| 0.85 | 0.276 | 0.974 | 0.889 | 0.644 |
| 0.90 | 0.069 | 1.000 | 1.000 | 0.591 |
| 0.95 | 0.000 | 1.000 |  | 0.574 |

supTable 7. Threshold‐dependent diagnostic performance of the EXT model for predicting 28-day mortality of severe pulmonary complications (SPCs) in the test cohort, showing sensitivity, specificity, positive predictive value (PPV), and negative predictive value (NPV) at different predicted probability cut-offs.
