# Supplementary material for: PRESCO: an online tool for predicting severe pulmonary complications and survival after cancer surgery
Source: Front Oncol. 2026 Jan 7;15:1705181. doi: 10.3389/fonc.2025.1705181 (PMC12819265; doi:10.3389/fonc.2025.1705181)
Supplement: Supplementary file 8 [file Table8.docx]

| **threshold** | **sensitivity** | **specificity** | **PPV** | **NPV** |
| --- | --- | --- | --- | --- |
| 0.05 | 1.000 | 0.065 | 0.561 | 1.000 |
| 0.10 | 0.973 | 0.226 | 0.600 | 0.875 |
| 0.15 | 0.973 | 0.387 | 0.655 | 0.923 |
| 0.20 | 0.919 | 0.484 | 0.680 | 0.833 |
| 0.25 | 0.892 | 0.581 | 0.717 | 0.818 |
| 0.30 | 0.892 | 0.677 | 0.767 | 0.840 |
| 0.35 | 0.865 | 0.806 | 0.842 | 0.833 |
| 0.40 | 0.838 | 0.839 | 0.861 | 0.812 |
| 0.45 | 0.838 | 0.871 | 0.886 | 0.818 |
| 0.50 | 0.838 | 0.903 | 0.912 | 0.824 |
| 0.55 | 0.784 | 0.903 | 0.906 | 0.778 |
| 0.60 | 0.730 | 0.935 | 0.931 | 0.744 |
| 0.65 | 0.730 | 0.935 | 0.931 | 0.744 |
| 0.70 | 0.730 | 0.935 | 0.931 | 0.744 |
| 0.75 | 0.676 | 0.935 | 0.926 | 0.707 |
| 0.80 | 0.649 | 0.935 | 0.923 | 0.690 |
| 0.85 | 0.595 | 0.968 | 0.957 | 0.667 |
| 0.90 | 0.432 | 0.968 | 0.941 | 0.588 |
| 0.95 | 0.270 | 1.000 | 1.000 | 0.534 |

supTable 8. Threshold‐dependent diagnostic performance of the RDF model for predicting 90-day mortality of severe pulmonary complications (SPCs) in the test cohort, showing sensitivity, specificity, positive predictive value (PPV), and negative predictive value (NPV) at different predicted probability cut-offs.
